# Evaluating best practice in informed consent discussions: a new method of evaluating information provision and patient understanding during trial recruitment consultations

**DOI:** 10.1186/1745-6215-14-S1-O70

**Published:** 2013-11-29

**Authors:** Julia Wade, Jenny Donovan, Sangeetha Paramasivan, Athene Lane, David Neal, Freddie Hamdy

**Affiliations:** 1 University of Bristol, Bristol, UK; 2 University of Cambridge, Cambridge, UK; 3 University of Oxford, Oxford, UK

## Background

Use of discussion and questioning is an effective supplement to written information provision during trial recruitment. Current evaluations of recruitment consultations monitor information delivered by recruiters, ignoring evidence of patient understanding/misunderstanding. This study a) developed a method of evaluating the quality of information provision and understanding during informed consent consultations for RCT recruitment (QUICC-RCT) that took patient responses into account and b) investigated the feasibility of applying it as a tool to evaluate informed consent (IC) in recruitment consultations.

## Methods

The QUICC-RCT, developed from qualitative research, used principles of conversation analysis regarding conversation structure and turn-taking to identify key parameters in recruiter and patient talk for combined quantitative and qualitative evaluation. Next, QUICC was applied to audio-recordings of 25 IC consultations from three trials, to evaluate whether patients were well-informed, accepting of randomisation and in equipoise at time of decision-making about trial participation.

## Findings

A flexible framework was developed in which a broad sweep analysis of the entire consultation could be followed by more detailed analyses of key sections as required, making it practical for application to diverse trial recruitment consultations. Preliminary findings suggest that evaluation of recruiter-patient interaction should focus on evidence of a) patient views on the trial, randomisation and treatments; b) improved patient understanding; and c) patient equipoise. The approach identifies recruiter behaviour that promotes good understanding. Findings from the full dataset will be presented.

## Conclusions

The QUICC-RCT enables evaluation of quality of recruiter communication and extent of patient understanding during trial recruitment consultations.

